# Structure of Simple Dipolar Water-Like Fluids: Primitive Model and Hard Tetrahedra

**DOI:** 10.3389/fchem.2021.783741

**Published:** 2021-12-07

**Authors:** I. Nezbeda

**Affiliations:** ^1^ Institute of Chemical Process Fundamentals, Czech Academy of Sciences, Prague, Czech Republic; ^2^ Faculty of Science, J. E. Purkinje University, Ústí nad Labem, Czech Republic

**Keywords:** hard tetrahedron fluid, primitive models of water, screened dipole-dipole interaction, structure of dipolar associating fluids, perturbed equations of state

## Abstract

Dipolar versions of two qualitatively different types of simple short range model fluids which exhibit the phenomenon of hydrogen bonding and which could thus serve as a reference in equations of state for associating fluids have been considered: the primitive model of water descending from the TIP4P model and the fluid of hard tetrahedra. The hydrogen bonding structure exhibited by the latter model results from purely repulsive interactions whereas in the first model the “hydrogen bonding interaction” is explicitly incorporated in the model. Since the water molecules bear a strong dipole moment, the effect of the added dipole-dipole interaction on the structure of the two short-range models is therefore examined considering them both in the full and screened dipole-dipole modifications. It is found that the hydrogen bonding structure in the primitive model resulting from the site-site interactions is so strong that the additional dipole-dipole interaction has only a marginal effect on its structure and contributes thus only to the internal energy. On the contrary, even only a weak dipole-dipole interaction destroys the original hydrogen bonding structure of the hard tetrahedron fluid; to preserve it, a screened dipole-dipole interaction has to be used in the equation of state development.

## 1 Introduction

Simple models have played a fundamental role in our understanding of molecular mechanisms governing the behavior of fluids and for the development of tractable expressions of their thermodynamic properties. The use of simple models goes as far back as to van der Waals (vdW) who used, intuitively, the notion of the excluded volume (hard body model) for the development of his famous equation of state (EoS). It took nearly one century before the vdW equation was laid on a rigorous theoretical footing using a perturbation expansion which is the only theoretical tool to deal with complex realistic interaction models. It is based on results of molecular simulations carried out during the 1960s and which showed that the structure of normal (i.e., non-polar) fluids can be well estimated by that of appropriate purely repulsive hard body fluids which is a necessary condition for the perturbation expansion about a hard body reference to converge (Barker and Henderson, 1976). Further extensive simulations in 1990’s and at the beginning of 2000’s then extended this finding also to complex fluids: It was shown that the structure of fluids is determined, in general, by *short-range* interactions which however may be not only repulsive (which is the case of normal fluids) but also attractive (in the case of polar and associated fluids); for a review see (Nezbeda, 2005). These findings have extended the potential of the perturbation expansion to derive a molecular-based EoS for the entire class of fluids and write such an EoS in the form

$$z\equiv \frac{\beta P}{\rho }=z_{\text{ref}}+\Delta z$$ (1)

which results from a decomposition of the considered intermolecular interaction model (force field), *u* (1, 2), into short-range reference, *u*_SSR_(1, 2), and perturbation, *u*_pert_ (1, 2), parts,

$$u\left(1,2\right)=u_{\text{SSR}}\left(1,2\right)+u_{\text{pert}}\left(1,2\right)$$ (2)

In the above equations symbol (1,2) stands for the complete set of generalized coordinates of molecules 1 and 2, *P* is the pressure, *β* is the inverse temperature, *β* = 1/*k*_B_ *T*, where *k*_B_ is the Boltzmann’s constant and *T* is the temperature, *ρ* is the number density, *ρ* = *N*/*V*, *z*_ref_ is the compressibility factor of the reference system, and Δ*z* is a correction term.

The only long-range part of *u* (1, 2) is the Coulombic interaction. In all so far performed simulations on the effect of the range of interactions, the SRR has been obtained by a gradual switching off this interaction (for a review see (Nezbeda, 2005)),

$$u_{\text{SSR}}\left(1,2\right)=u\left(1,2\right)-S\left(r_{12};R^{\prime },R^{\prime \prime }\right)u_{\text{Coul}}\left(1,2\right)$$ (3)

where *S* is a switch function,

$$S\left(R^{\prime },R^{\prime \prime };r\right)=\left\{\begin{array}{cc} 0 & for\;r<R^{\prime }\\ {\left(r-R^{\prime }\right)}^{2}\left(3R^{\prime \prime }-R^{\prime }-2r\right)/{\left(R^{\prime \prime }-R^{\prime }\right)}^{3}\; & for\;R^{\prime }<r<R^{\prime \prime }\\ 1\; & for\;r>R^{\prime \prime } \end{array}\right.,$$ (4)

*r*_12_ is the distance between reference sites of the molecules, and *R*′ and *R* ^″^ are its appropriately chosen parameters (for details of the effect of various choices of *R*′ and *R* ^″^ see (Nezbeda and Kolafa, 1999)).

In addition to the constraint that the reference fluid has to reproduce the structure of the considered fluid, the choice of the reference is also subject to another constraint: the reference has to be amenable to a theoretical treatment resulting in close analytic expressions for its properties. And this is the point where simple models enter the process: The fluid defined by potential (3) remains too complex to provide analytic results and simple models are therefore used to approximate its properties.

**FIGURE 1:**
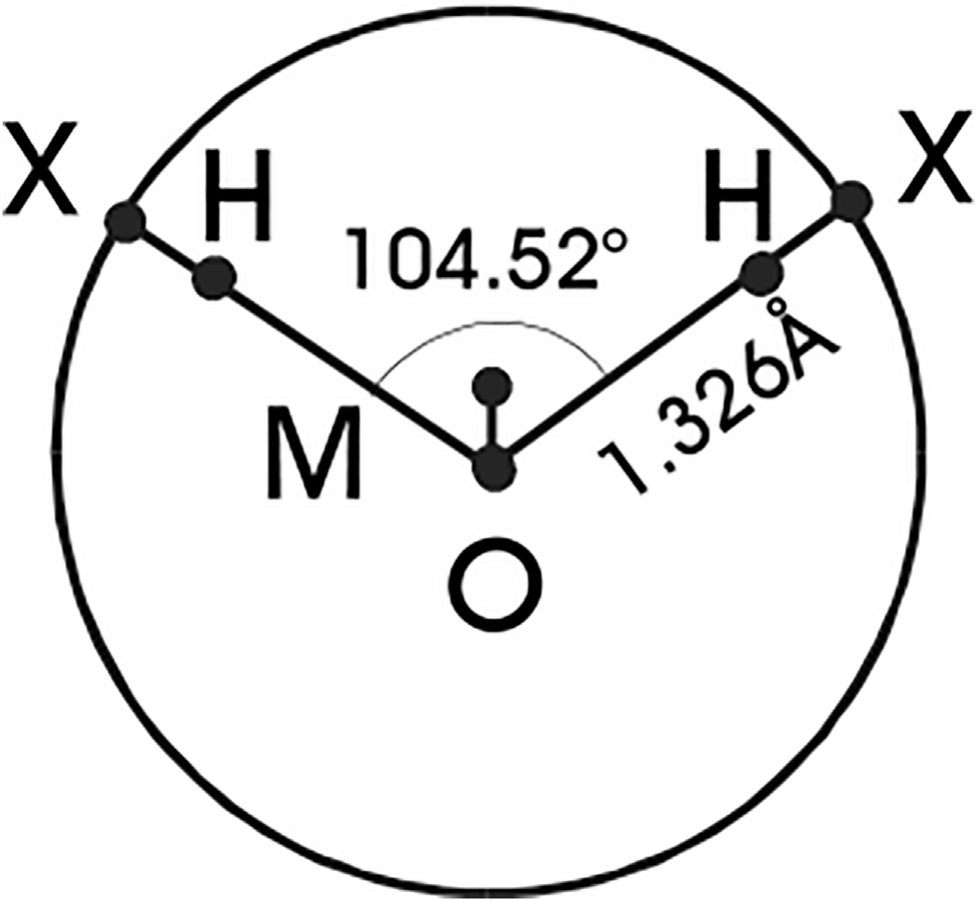
Schematic representation of the TIP4P geometry of water molecules and the primitive model. Sites X represent the hydrogen-like sites H moved on the surface of the oxygen sphere.

Confining our considerations henceforth to associating fluids exemplified by water, its primary distinctive feature is its open structure resulting from hydrogen bonding (H-bonding) and this phenomenon has to be reproduced by any simple model considered for the reference in the perturbation approach. First such simple intuitive models emerged in the end of the 1980’s (BoL, 1982; Dahl and Andersen, 1983; Smith and Nezbeda, 1984; Kolafa and Nezbeda, 1987). All of them employ a hard sphere with embedded attractive sites, using typically a square-well interaction, and different models differ then in the number of the H-bonding sites and their geometry. Later on, such models (referred to as primitive models, PM) have been linked directly to realistic force fields (parent model) and constructed as their descendants using statistical mechanical tools (Nezbeda, 1997; Vlcek and Nezbeda, 2004a), see Figure 1. Essentially the same models, but without any link to realistic force fields, have also been used in SAFT equations (Vega and Llovell, 2016).

Besides the PM’s where the H-bonding structure is forced explicitly by the attractive site-site interactions, a hard body model which also exhibits a water-like structure is the fluid of hard tetrahedra (Kolafa and Nezbeda, 1995). This model is therefore another possible simple model to be used as a reference both in a perturbed molecular-based EoS or in a vdW-like EoS for water. This choice may be viewed as an associated fluid counterpart of hard spheres (hard body) used for non-polar fluids. Its properties have been the subject of intensive research (Haji-Akbari et al., 2011; Kolafa and Labík, 2015; Irrgang et al., 2017) and an EoS in a close analytic form is also available (Tian et al., 2019).

In order to complete the perturbed EoS development it is necessary to consider contributions of the Coulombic interactions screened at short separations,

$$u_{\text{pert}}\left(1,2\right)=S\left(r_{12};R^{\prime },R^{\prime \prime }\right)u_{\text{Coul}}\left(1,2\right)$$ (5)

Since water molecules possess a strong dipole moment, in practical computations the leading term of the electrostatic interactions is (very accurately) approximated by the leading dipole-dipole interaction, *u*_DD_ (1, 2). In the evaluation of Δ*z*_DD_ one should however bear in mind that, according to Eq. 5, the DD interaction should be considered only over a reduced intermolecular separations to avoid its double counting at close separations. This has been the case of molecular-based equations (Nezbeda and Pavlíček, 1996; Jirsák and Nezbeda, 2007a) but not of vdW-like equations for water (Vega and Llovell, 2016). Formally, the omission of this constraint may not cause problems. At the macroscopic level it may improve the performance of the equations but, on the other hand, it also may turn such an EoS to a purely empirical one and at the molecular level such an equation may thus lose any justification.

The primary motivation for this study has been the examination of the effect of adding the dipole-dipole interaction, either completely into the reference or in its screened form only, on the structure and, consequently, on the EoS development. We consider therefore two qualitatively different simple model fluids, i) the dipolar primitive model of water descending from the TIP4P (Jorgensen et al., 1983) force field with the explicit site-site interactions producing the H-bonding structure, and ii) the fluid of dipolar hard tetrahedra which itself, without the DD interaction, adopts an H-bonding-like structure solely due to purely repulsive interactions (Kolafa and Nezbeda, 1995). The dipole-dipole interaction is considered both over the entire range and screened at short separations. Carrying out standard Monte Carlo simulations we focus on the structure described by the complete set of the site-site correlation functions. In the next Section we provide necessary theoretical and computational details and in the following Section results are presented and discussed. The main findings are summarized in the last Section along with a potential further development.

## 2 Basic Definitions and Computational Details

### 2.1 The Models

We are going to consider two types of short-range dipolar hard-core models, the fluid of hard tetrahedra and the primitive model of water descending from the realistic TIP4P model (Jorgensen et al., 1983).

#### 2.1.1 Primitive Model of Water

Primitive models descend, in general, from realistic models by neglecting all medium (van der Waals) and long ranged (electrostatic) interactions, and account qualitatively only for the short-range, both repulsive and attractive interactions.

The parent TIP4P models have, in addition to the central uncharged oxygen site, two H-like sites bearing a positive charge and an M-site with a negative charge, see Figure 1. Denoting the sites which bear charges as P (positive charge) and N (negative charge), and the site without electrostatic interactions as O, the complete intermolecular interaction energy of the PM is given by [fore details see (Vlcek and Nezbeda, 2004a)].

$$u_{\text{PM}}\left(1,2\right)=u_{\text{HS}}\left(r_{\text{OO}};d_{ij}\right)+\underset{i=j}{\underset{i,j\in \left\{P,N\right\}}{\sum }}u_{\text{HS}}\left(|r_{i}^{\left(1\right)}-r_{j}^{\left(2\right)}|;d_{ij}\right)+\underset{i\neq j}{\underset{i,j\in \left\{P,N\right\}}{\sum }}u_{\text{SW}}\left(|r_{i}^{\left(1\right)}-r_{j}^{\left(2\right)}|;R_{\text{SW}}\right),$$ (6)

where the summation in the second term of this equation runs over the pairs of like sites, in the third term over the pairs of unlike sites,

$$\begin{array}{cccc} u_{\text{HS}}\left(r_{12};\sigma \right) & = & +\infty , & \text{for }r_{12}<\sigma \\ & = & 0, & \text{for }r_{12}>\sigma , \end{array}$$ (7)

and

$$\begin{array}{cccc} u_{\text{SW}}\left(r_{12};R_{\text{SW}}\right) & = & -\epsilon_{\text{HB}}, & \text{for }r_{12}<R_{\text{SW}},\\ & = & 0, & \text{for }r_{12}>R_{\text{SW}}. \end{array}$$ (8)

#### 2.1.2 Hard Tetrahedron Fluid

**FIGURE 2:**
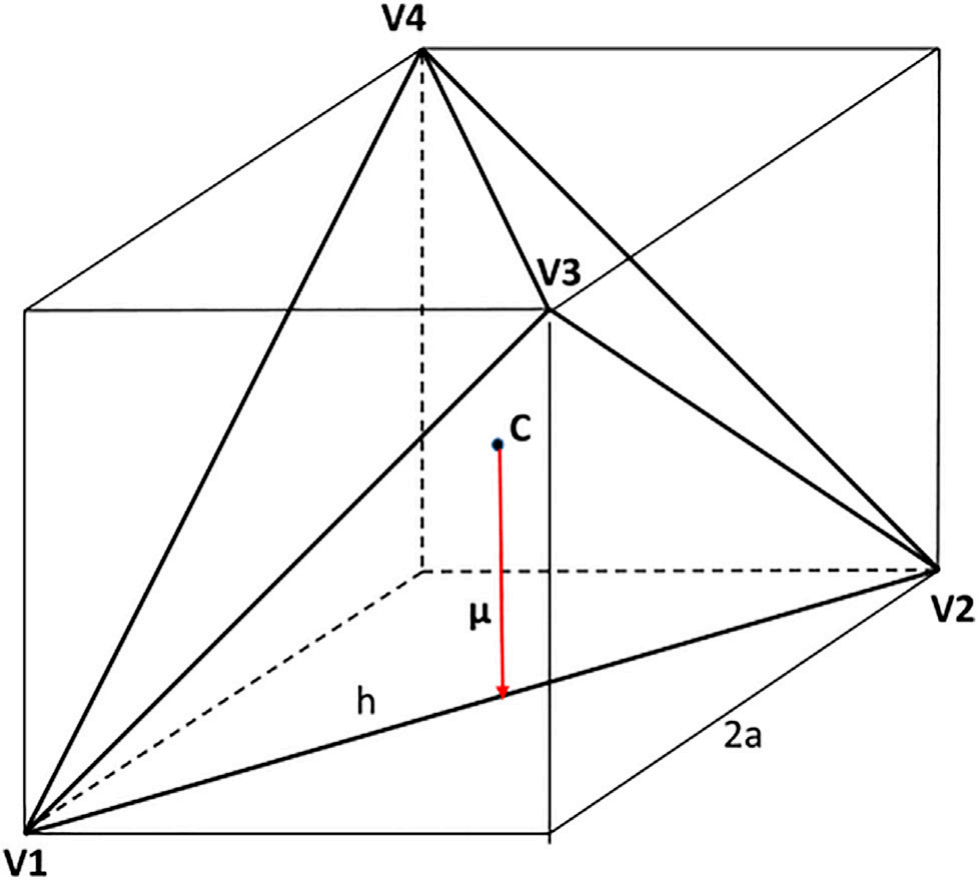
Hard tetrahedron with the dipole moment (red arrow).

There are different conventions, and hence also different scalings, used to describe the geometry of tetrahedra, see Figure 2. Kolafa and Nezbeda (Kolafa and Nezbeda, 1995), and also Haji-Akbari et al. (Haji-Akbari et al., 2011), considered a regular tetrahedron inscribed in a cube of the edge length equal to 2*a* whereas Kolafa and Labik (Kolafa and Labík, 2015) then used tetrahedron’s edge, *h*, to scale distances. For convenience, and to keep contact with the fluid of hard spheres (HS), we scale in this paper distances by diameter *σ*_HS_ of the circumscribed sphere. Thus, $a=\left(\sqrt{3}/6\right)\sigma_{\text{HS}}$ , $h=\sqrt{\left(2/3\right)}\sigma_{\text{HS}}$ , and the tetrahedron’s volume is $V=\left(\sqrt{3}/27\right)\sigma_{\text{HS}}^{3}$ . Hard tetrahedron interaction potential is thus given by.

$$\begin{array}{c} u_{\text{HT}}\left(1,2\right)\;=\;+\infty \;\text{for}R_{12}<\sigma_{\text{HS}}/3 \end{array}$$ (9)

$$\begin{array}{c} =\;+\infty \;\text{for}\sigma_{\text{HS}}/3<R_{12}<\sigma_{\text{HS}}\;\text{when overlap occurs} \end{array}$$ (10)

$$\begin{array}{c} =\;0\;\text{for}\sigma_{\text{HS}}/3<R_{12}<\sigma_{\text{HS}}\;\text{when no overlap occurs} \end{array}$$ (11)

$$\begin{array}{c} =\;0\;\text{for}\;R_{12}>\sigma_{\text{HS}} \end{array}$$ (12)

where *R*_12_ denotes the center-to-center separation. The structure of this fluid was discussed in (Kolafa and Nezbeda, 1995) and the virial coefficients and an EoS are also available (Kolafa and Labík, 2015; Irrgang et al., 2017; Tian et al., 2019).

#### 2.1.3 Dipolar Models

Dipolar versions of the above models are obtained by adding a point dipole of strength *μ* to the central site,

$$u_{\text{DD}}\left(1,2\right)=\mu^{2}\left[3\left(e_{1}\cdot {\hat{R}}_{12}\right)\left(e_{2}\cdot {\hat{R}}_{12}\right)-\left(e_{1}\cdot e_{2}\right)\right],$$ (13)

where ${\hat{R}}_{12}$ is a unit vector in the direction of center-to-center vector **R**_12_, and **e**_*i*_ is a unit vector in the direction of dipole *i*. The total interaction potential of the considered models assumes thus the form

$$u_{}(1,2)=u_{\text{ref}}\left(1,2\right)+u_{\text{DD}}\left(1,2\right)$$ (14)

where *u*_ref_ (1, 2) is either *u*_HT_ (1, 2) or *u*_PM_(1, 2). However, as it is clear from Eq. 3, the formal addition of the dipole-dipole interaction to models which already include H-bonding is not fully correct; the dipole-dipole interaction has to be suppressed and the dipolar models should thus properly possess this modified form,

$$u_{}(1,2)=u_{\text{SRR}}\left(1,2\right)+S\left(r_{\text{OO}};R^{\prime },R^{\prime \prime }\right)u_{\text{DD}}\left(1,2\right)$$ (15)

Dipolar hard body fluids are characterized by a dimensionless energetic parameter *λ* which combines the strength of the dipole-dipole interaction with the scaling parameter,

$$\lambda =\frac{\mu^{2}}{k_{\text{B}}T\sigma_{\text{HS}}^{3}},$$ (16)

where *β* = 1/*k*_B_ *T*, *k*_B_ is the Boltzmann’s constant and *T* is the temperature. The direction of the dipole moment in the PM is defined by sites O and M. Concerning hard tetrahedra, there is no a priori reason for a specific direction of tetrahedron’s dipole moment. In (Kolafa and Nezbeda, 1995) it was argued that the face-to-face packing of tetrahedra in dense systems resembles a hydrogen bond network in water and a geometric definition of a “hydrogen bond” was provided [for further details see (Kolafa and Nezbeda, 1995)]. In this view the vertices of tetrahedron may be identified with the location of two hydrogen atoms and the lone electron pair (TIP5P-type geometry). We thus define the dipole moment as the vector from the center of the tetrahedron to the center of an tetrahedron’s edge.

### 2.2 Simulation Details

For simulations with the site-site models it is convenient to follow the physical picture and represent the dipole by two charged sites with charges *q*_*i*_ = ±1 a distance *l*_0_ apart; the dipole moment is then *μ* = *ql*_0_. The closest approach of two PM molecules is *σ*_HS_ and the value *l*_0_/*σ*_HS_ was set to 0.01. The closest approach of two tetrahedra is *σ*_HS_/3 and a lower value of *l*_0_ had therefore to be used, *l*_0_/*σ*_HS_ = 0.001. Test simulations were performed to ensure that the use of these values yielded, within the simulation errors, the same result as those with the point dipole.

To account for the long ranged character of the Coulombic interactions, the reaction field method (Neumann et al., 1984) was employed. The dipole-dipole interaction energy assumed thus the form.

$$\begin{array}{c} \beta u_{\text{DD}}=\epsilon \underset{i\in \left\{a\right\},j\in \left\{b\right\}}{\sum }\frac{q_{i,a}q_{j,b}}{\left|r_{i,a}-r_{j,b}\right|}\left[1+\frac{\varepsilon_{r}-1}{2\varepsilon_{r}+1}{\left(\frac{\left|r_{i,a}-r_{j,b}\right|}{r_{c}}\right)}^{3}\right]\;for\;R_{12}<r_{c} \end{array}$$ (17)

$$\begin{array}{c} =0for\;R_{12}>r_{c} \end{array}$$ (18)

where the summation runs over the charges on molecules *a* and *b*, the second term is the reaction field correction for the finite radius cutoff, *r*_*c*_, *ɛ*_*r*_ is the dielectric constant of the surrounding continuum set to infinity in the simulations, and *ϵ* is related to the conventional parameter *λ* by the relation

$$\epsilon =\lambda /l_{0}^{2}$$ (19)

Standard simulations in an NVT ensemble (Frenkel and Smit, 2002) with 512 particles were performed at several densities and for a number of values of *λ*. Control quantities (Nezbeda and Kolafa, 1995) were always computed and the evolution of the energy was monitored to follow the development of the system to be sure that the productive runs started from a properly equilibrated configuration. The structure of the considered fluids was the primary quantity of interest in this study. All necessary information is provided by the full pair correlation function *g* (1, 2) whose complete determination is however practically impossible. The usual way is to characterize the structure of fluids by a set of partially averaged, i.e., the site-site correlation functions *g*_*ij*_ (Nezbeda and Smith, 1981),

$$4\pi \;r_{ij}^{2}\;g_{ij}\left(r_{ij}\right)=\left(1-\frac{1}{N}\right)V\left\langle \delta \left(r_{ij}-|r_{1}^{\left(i\right)}-r_{2}^{\left(j\right)}|\right)\right\rangle ,$$ (20)

where *δ* is the Dirac delta distribution, and ⟨…⟩ denotes an ensemble average. Another quantity of interest obtainable from the center-to-center correlation function, *g*_CC_, is the coordination number,

$$N_{\text{C}}=4\pi \rho \int_{0}^{R_{\text{min}}}g_{\text{CC}}\left(r\right)r^{2}\text{d}r$$ (21)

which differentiates associating fluids from non-polar ones; *R*_min_ in this equation denotes the location of the first minimum of *g*_CC_. Low values of the coordination number (the number of molecules in the 1st coordination shell) within the range from 4 to 6 at liquid densities point to a typical water-like structural arrangement in the liquid.

## 3 Results and Discussion

Using the methodology described in the preceding section we performed simulations for a series of values of parameter *λ* with the switching function whose parameters were taken from the previous studies (Nezbeda and Kolafa, 1999; Kolafa and Nezbeda, 2000) and for a series of densities with the main focus on the structure described by the complete set of the site-site correlation functions.

When setting the parameters of the dipolar models we wanted to keep contact with real water. The dipole moment of the water molecule (gas value) is 1.85D. The size of the water molecule is not uniquely defined but its value used in various applications varies around 3Å. Confining the considerations to the room temperature we get then for parameter *λ* value around 3. When the liquid value of *μ* is used, *μ* ≈ 2.9D, then *λ* is around 7. For comparison, in their extensive study of the dipolar HS fluid Theiss and Gross (Theiss and Gross, 2019) reported thermodynamic functions up to *λ* = 7, which was also the upper limit in our simulations.

### 3.1 Primitive Model

All parameters of the PM but the site-site strength, *ϵ*_SW_, are obtained from the parent TIP4P model using statistical mechanical tools and taken from (Vlcek and Nezbeda, 2004a). For *ϵ*_SW_/*k*_B_ *T* Jirsak and Nezbeda (Jirsák and Nezbeda, 2007b) used the value 4440 K to reproduce the temperature density maximum. Another possibility is to consider the value 3300 K which yields the experimental energy of the dimer. In theoretical study (Vlcek and Nezbeda, 2004b), Vlcek and Nezbeda reported results for the PM up to *β* ≡*ϵ*_SW_/*k*_B_ *T* = 8. Since relating the PM to the full water model is not, in principle, correct (Nezbeda, 2020) and because of ambiguity of its value, we decided to use also the lowest temperature, *β* = 8. As regards density, as a consequence of its relatively open structure, liquid water packing fraction is lower than that of, e.g., argon-like fluids. Majority of simulations were therefore carried out at a typical density, *η* = 0.35, and these results are also reported here.

In previous studies on the effect of the long-range interactions on the structure of water (Nezbeda and Kolafa, 1999; Kolafa and Nezbeda, 2000) it was shown that the shortest cutoff guaranteeing the identity of the structures was the range $<4\overset{{}^{\circ}}{A},6\overset{{}^{\circ}}{A}>$ and the same values were therefore used also for *R*′ and *R* ^″^ using for the HS diameter the same value, i.e., *σ*_HS_ = 2.653Å.

**FIGURE 3:**
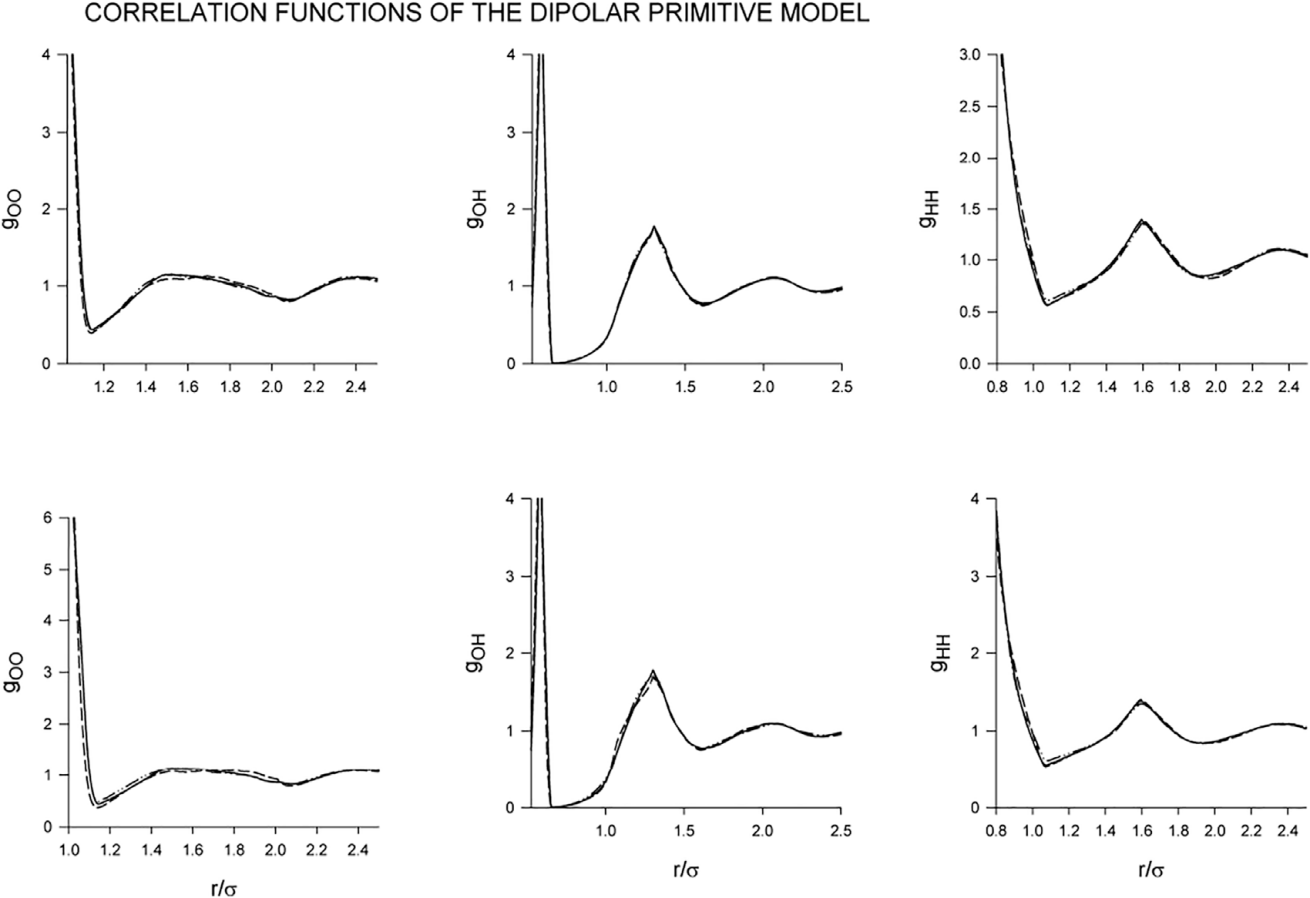
Dependence of the correlation functions of the dipolar primitive model on the gradual inclusion of the dipole-dipole interaction for *λ* =3 **(upper row)** and *λ* =7 **(lower row):** Full line (no dipole), short-dashed line (screened dipole), long-dashed line (full dipole). The results for the non-polar and screened models are not distinguishable within the scale of the graph.

Changes in the structure in dependence on *λ* and the gradual inclusion of the dipole-dipole interaction at the given conditions are shown in Figure 3. As we see, the effect of the dipole-dipole interaction does not seems, perhaps surprisingly, significant with the results of the screened and zero DD interaction being barely distinguishable. The only noticeable difference we find in *g*_OO_ for *λ* = 7. The strong dipole-dipole attraction increases the population around the central particle but this effect has only a marginal impact on the coordination number which in all cases attains values about 3.95. Concerning the site-site correlation functions, *g*_OH_ and *g*_HH_, they seem to remain intact so that also the orientational arrangement is not affected by the added dipole-dipole interaction. Consequently, although the chosen temperature, *β* = 8, is very low and corresponding to cold liquid water, the resulting H-bonding is so strong that the added dipole-dipole interaction does not seem to be able to break it.

Unlike the structure, the additional interaction has to have an impact on the energy which is shown in Table 1. The contribution of the dipole-dipole interaction is rather small, perhaps also surprisingly small, and becomes comparable with the H-bonding energy only after the full inclusion (no screening) for the highest value of *λ*, *λ* = 7.

**TABLE 1:** The total energy, *βE*/*N*, of the dipolar primitive model for different dipole-dipole interactions.

Model	*λ* = 3	*λ* = 7
no dipole	−1.727	−1.727
screened dipole	−1.983 ± 0.009	−2.62 ± 0.01
full dipole	−2.445 ± 0.006	−3.43 ± 0.01

### 3.2 Fluid of Hard Tetrahedra

**FIGURE 4:**
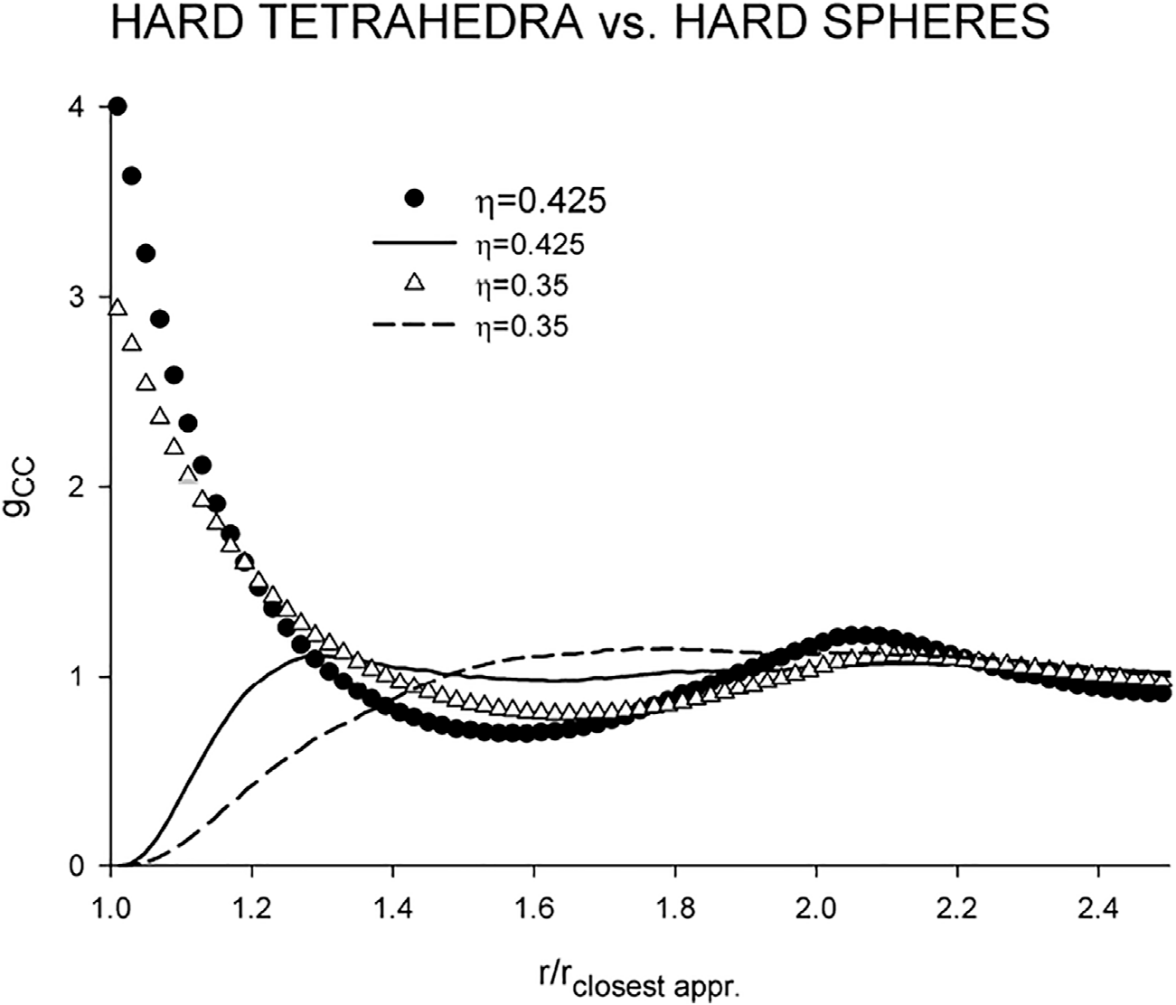
Comparison of the center-center correlation functions of the hard tetrahedron fluid (lines) with those of the hard sphere fluid (symbols).

Despite its simple and compact shape, hard tetrahedron is an extremely nonspherical body. For comparison, its convex body shape factor *α* is 2.2346 which corresponds, e.g., to hard prolate spherocylinders of the length-to-breadth ratio greater than 5 (Boublík and Nezbeda, 1986). It is therefore instructive to compare first the center-center correlation functions of the fluids of hard spheres and tetrahedra at different densities; see Figure 4 where the distances are scaled by the respective closest approach distance.

**FIGURE 5:**
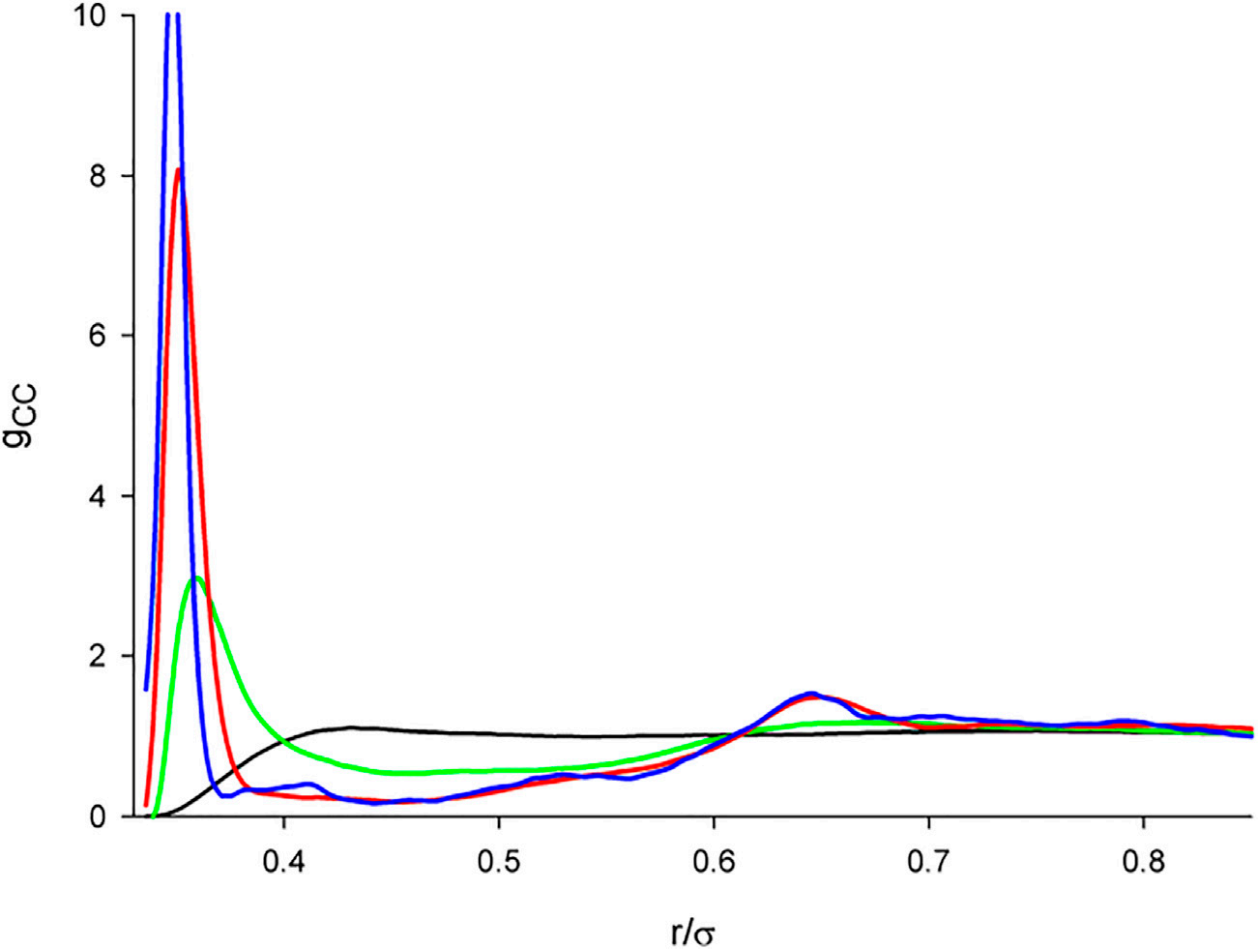
The center-center correlation functions of the dipolar hard tetrahedron fluid in dependence on the dipole-dipole interaction strength. *λ* = 0 (black); *λ* = 0.5 (green); *λ* = 1.0 (red); *λ* = 0 (blue).

*g*_cc_ of the HS fluid remains qualitatively the same and changes only numerically with increasing density with the coordination number remaining also the same, about 12, which is a typical number for nonpolar fluids, and the same applies also to the location of the minimum, *r*/*r*_closest appr_ ≈ 1.6. *g*_cc_ of the HT fluid is rather flat without a pronounced first peak (cf. Figure 5 of (Kolafa and Nezbeda, 1995)). Although density *η* = 0.35 falls, generally, into a higher medium liquid density range, the HT fluid at this density still behaves as a rare gas with the coordination number about 20. Structural changes thus take place over a relatively narrow range of high densities. At packing fraction *η* = 0.425, which is close to the onset of metastability (Kolafa and Labík, 2015), the minimum of *g*_CC_ moves to the same location as that of the HS fluid, *r*/*r*_closest appr_ ≈ 1.6, and the coordination number drops to about 3. With respect to the above findings, namely that the hard tetrahedron fluid at medium density behaves as rare gas, we will present and discuss its dipolar versions only for the high density. As regards values of *λ*, a direct comparison between *λ*_tetrahedron_ and *λ*_HS_ is not possible. It is necessary to realize that the latter is scaled by the closest approach which is about three times larger than that for tetrahedra. The preferably used value in this paper, *λ* = 1, represents therefore a very strong dipole-dipole interaction.

To examine the effect of the dipole-dipole interaction and its screening, it is also necessary to set the parameters of the switching function. In the case of the PM, the dipole-dipole interaction was completely switched off directly at the end of the hard core repulsion (*r*/*σ*_HS_ = 1) but at a distance, approximately, *R*′/*σ*_HS_ = 1.5 away with a relative width of the switching range 0.75. With no other information on the switching available, the same values have been used therefore as a guidance for setting the same range, $<R^{\prime },R^{\prime \prime }>$ , also for the tetrahedron fluid.

The water-like arrangement of tetrahedra is exclusively only due to their shape so that it is relatively fragile and it may be thus expected that any additional (perturbing) interaction may destroy it. To confirm this intuitive presumption, we show in Figure 5 the center-center correlation functions, *g*_CC_, for a series of *λ*. As it is seen, by adding a dipole, regardless of its strength, the structure of the fluid changes. In fact, the *g*_CC_ function strongly resembles that of the dipolar HS characterized by a very high and narrow peak close to the contact (cf. Figure 4 of (Ganzenmüller and Camp, 2007)). The minimum also moves towards contact and then *g*_CC_ becomes rather flat with an indistinctive second maximum, unlike the HS case which continues to exhibit sharp peaks at multiples of the HS diameter. The coordination number drops down to about 2. In contrast to the HS case, for hard tetrahedra away from the contact the DD interaction competes with their shape which suppresses the exclusive DD interaction dominance and hence also no further sharp peaks are observed.

**FIGURE 6:**
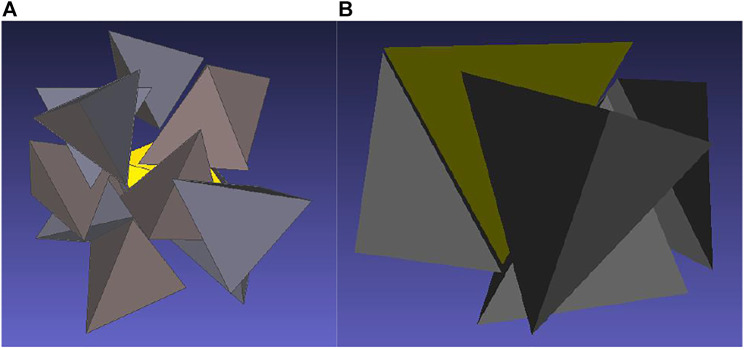
Visualization of the arrangement of tetrahedra around a central tetrahedron at *η* = 0.425 for nonpolar tetrahedra **(A)** and for dipolar tetrahedra with *λ* = 1 **(B)**.

The low coordination number deserves a discussion. An analysis of configurations reveals that, typically, two polar tetrahedra are arranged face-to-face around the central tetrahedron which explains the very high peak of *g*_CC_ close to contact, see Figure 6B. There can we also see two other tetrahedra in vicinity of the central body which would mean that there are (may be) four molecules around the central one which would correspond to the structure of low temperature water. However, the two tetrahedra are oriented with their vertices pointing to the central tetrahedron so that their center falls slightly beyond the range determined by the minimum of the correlation function *g*_CC_. It thus seems that the definition of the coordination shells based on the minima of the center-center correlation function, which suits well for systems made up of not too nonspherical molecules, may be misleading for highly nonspherical molecules as, e.g., hard tetrahedra: some tetrahedra may fall into vicinity of the central tetrahedron by their vertex but their center may be then outside the range. For comparison we show in Figure 6A the surrounding of a nonpolar tetrahedron. As mentioned above, a typical gas-like chaotic distribution is observed.

**FIGURE 7:**
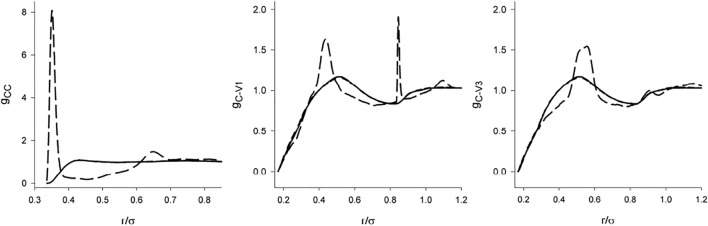
The center-center/site correlation functions of the hard tetrahedron fluid of *λ* = 1 in dependence on the range of the interaction: solid line (no dipole), short-dashed line (screened dipole), short-dashed line (full dipole). The results for the non-polar and screened models are not distinguishable within the scale of the graph.

**FIGURE 8:**
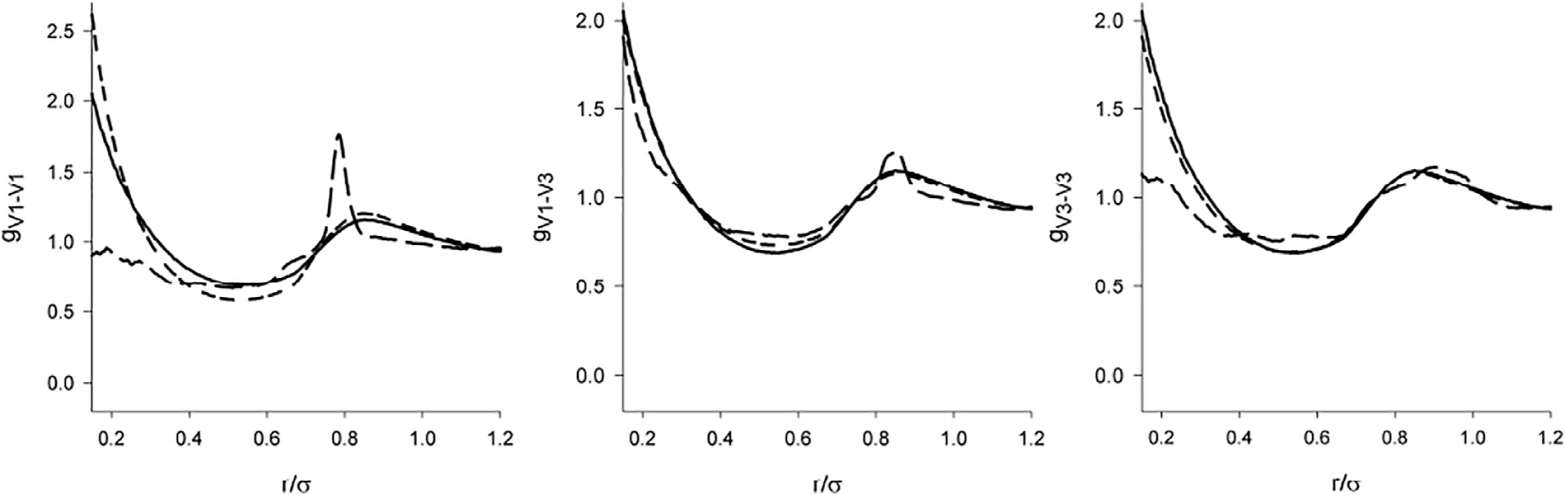
The same as Figure 7 for the vertex-vertex correlation functions.

Information on the orientational arrangement is provided by the complete set of the site-site correlation functions *g*_CV_ and *g*_VV_. These functions, both with the screened and full DD interaction for *λ* = 1 are shown in Figure 7 and Figure 8. Since the model with the dipole moment is asymmetric, the vertices are not equivalent. Denoting the edge connecting the charged vertices as 1-2 and the other two vertices as 3 and 4, then there are two different center-vertex correlation functions, [C-1] and [C-3], and 3 vertex-vertex correlation functions, (Barker and Henderson, 1976), (Barker and Henderson, 1976; Nezbeda, 2005; Nezbeda and Kolafa, 1999), and (Nezbeda and Kolafa, 1999).

There are two features of *g*_CV_ and *g*_VV_ which catch attention. First, in the full agreement with the claim that the structure is determined primarily by the short range interactions, we see that the dipole-dipole interaction switched on *beyond* the repulsive range has only a marginal effect. The C-V functions are nearly identical and the differences in the V-V functions are nearly negligible. Second, it is a relatively weak effect of the full DD interaction on the orientational arrangement (V-V functions). Except for the localized (and some sharp) maxima resulting from the interplay between the DD interaction and hard core geometry [e.g., hard tetrahedra *g* exhibits a cusp at $r/\sigma =\sqrt{\frac{2}{3}}$ ] the correlation functions of the full and screened models follow practically the same course. The full DD interaction is even suppressed at short separations for the V1-V1 and V3-V3 pairs.

## 4 Conclusion

Simulations of the dipolar versions of the two short-range models, the primitive model of water and hard tetrahedron fluid, have revealed differences in their response to adding the dipole-dipole interaction.

The primitive model, which has built-in hydrogen bonding, seems nearly “immune” to this additional interaction. It means, the dipole-dipole interaction does not cause practically any change in the structure of the liquid. This is a very favorable/important finding from the point of applications. Despite its long range, the dipolar version of the primitive model can be used directly as a suitable reference for the development of a molecular-based EoS. In the vdW-like approach the inclusion of the full Δ*z*_DD_ term represents then an acceptable approximation to the (theoretically correct) screened interaction. Since such a dipolar reference would capture most of the interactions and their contribution, it is expected that its amendment by a contribution of dispersion forces (Aim and Nezbeda, 1983) could yield, in both approaches, a good equation of state for water.

Unlike the primitive models, the hydrogen bonding structure exhibited by the hard tetrahedron fluid results from the purely excluded volume effect and is thus sensitive to any other added interaction. As discussed in the preceding section, the dipolar tetrahedron fluid may exhibit rather a complex behavior different from that of the non-polar tetrahedron model and its formal use as a reference would be thus erroneous. However, when the dipole-dipole interaction is screened, the structure of this model fluid does not change and it may be thus also used as a reference for the development of a molecular-based equation of state.

The last finding opens a possibility to follow the original vdW way of thinking which has resulted in plethora of commonly used cubic equations (Valderrama, 2003). These equations, with the hard sphere reference term, have been extended also to associating fluids by adding an associating term. The resulting equations are referred to as Cubic-Plus-Association (Yakoumis et al., 1998). This purely empirical approach may be cast into a physically acceptable/correct one if the hard sphere term is replaced by that which reflects H-bonding. The hard tetrahedron fluid may serve this purpose and such an attempt will the subject of future investigation.

## Data Availability

The raw data supporting the conclusion of this article will be made available by the author, without undue reservation.
